# Synergistic effect of simvastatin and ezetimibe on lipid and pro-inflammatory profiles in pre-diabetic subjects

**DOI:** 10.1186/1758-5996-2-34

**Published:** 2010-06-07

**Authors:** Ana-Lucia A Kater, Marcelo C Batista, Sandra RG Ferreira

**Affiliations:** 1 Division of Endocrinology, Internal Medicine Department, Federal University of São Paulo, Brazil; 2 Division of Nephrology, Internal Medicine Department, Federal University of São Paulo, Brazil; 3 Department of Nutrition, School of Public Health University of Sao Paulo, Brazil

## Abstract

**Background:**

Ezetimibe specifically blocks the absorption of dietary and biliary cholesterol and plant sterols. Synergism of ezetimibe-statin therapy on LDL-cholesterol has been demonstrated, but data concerning the pleiotropic effects of this combination are controversial.

**Objective:**

This open-label trial evaluated whether the combination of simvastatin and ezetimibe also results in a synergistic effect that reduces the pro-inflammatory status of pre-diabetic subjects.

**Methods:**

Fifty pre-diabetic subjects were randomly assigned to one of 2 groups, one receiving ezetimibe (10 mg/day), the other, simvastatin (20 mg/d) for 12 weeks, followed by an additional 12-week period of combined therapy. Blood samples were collected at baseline, 12 and 24 weeks. RESULTS: Total cholesterol, LDL-cholesterol and apolipoprotein B levels decreased in all the periods analyzed (p < 0.01), but triglycerides declined significantly only after combined therapy. Both drugs induced reductions in C-reactive protein, reaching statistical significance after combining ezetimibe with the simvastatin therapy (baseline 0.59 ± 0.14, simvastatin monotherapy 0.48 ± 0.12 mg/dL and 0.35 ± 0.12 mg/dL, p < 0.023). Such a reduction was independent of LDL-cholesterol change. However, mean levels of TNF-α and interleukin-6 and leukocyte count did not vary during the whole study.

**Conclusion:**

Expected synergistic lowering effects of a simvastatin and ezetimibe combination on LDL-cholesterol, apolipoprotein B and triglycerides levels were confirmed in subjects with early disturbances of glucose metabolism. We suggest an additive effect of this combination also on inflammatory status based on the reduction of C-reactive protein. Attenuation of pro-inflammatory conditions may be relevant in reducing cardiometabolic risk.

**Title/ID of trial registration:**

Effect of simvastatin and ezetimibe on lipid and inflammation/NCT01103648.

## Introduction

Long-term benefits of statins on primary and secondary prevention of cardiovascular events have been consistently shown in several populations [1-3]. It has been demonstrated that, with particular regard to subjects at high risk, the lower LDL-cholesterol levels, the lower the incidence of cardiovascular outcomes [4,5]. Evidence indicates that the beneficial effects of statins can be attributed to their lipid-lowering ability as well as to additional benefits. The so-called pleiotropic effects on low grade inflammation status have been described in subsets of subjects with different cardiovascular profiles [6,7]. The most common inflammatory marker used in clinical practice is the high-sensitivity C-reactive protein (CRP) level, but a number of others have also been investigated.

Disturbances of glucose metabolism accompanied by insulin resistance are pro-inflammatory conditions which may accelerate atherosclerotic process. Diabetic populations are considered at high cardiovascular risk and strict control of lipoprotein concentrations is recommended [8,9]. Several recent studies showed the efficacy of statins on primary and secondary prevention of cardiovascular events in diabetic populations [3,10,11]. The goal of 100 mg/dl for LDL-cholesterol may be too high for subjects at very high risk for whom a target of 70 mg/dL has been suggested [8,9]. To achieve this goal, high statin doses may be necessary, which increases its adverse effects. Given that statin monotherapy may be insufficient for the desirable reduction in LDL levels, a combination of lipid-lowering agents has become frequent in clinical practice. In particular, statin and ezetimibe combination has been shown to be very effective in reducing total and LDL-cholesterol levels [12,13].

Ezetimibe is a specific cholesterol absorption inhibitor that acts at the brush border of the small intestine, blocking the absorption of dietary and biliary cholesterol and plant sterols, resulting in intracellular cholesterol depletion via the Niemann-Pick C1-like transporter [14]. Adding ezetimibe to statin therapy induces a 15% reduction in LDL levels compared with only 6% achieved by doubling the dose of statins [15,16]. Data concerning the pleiotropic effects of this combination are controversial. One study, in which CRP level was used as the inflammatory marker, found that a combination of simvastatin and ezetimibe produced an incremental effect in lowering CRP, independently of the improvement in lipoprotein concentrations [17]. Although few studies have confirmed this finding [18-20], as far as we know, data regarding simvastatin-ezetimibe combination induced-changes in serum interleukin-6 (IL-6) and tumor necrosis factor alpha (TNF-α) levels are lacking. We tested the hypothesis that this combination would induce improvement in inflammatory status, as reflected by leukocyte count and CRP, IL-6 and TNF-α levels.

Therefore, this study evaluates whether the combination of lipid-lowering effects of low-to-moderate dose of simvastatin and ezetimibe also results in a synergistic effect that reduces the pro-inflammatory status of pre-diabetic subjects with mild-to-moderate hypercholesterolemia.

## Subjects and Methods

Participants were selected from the Federal University of São Paulo outpatient clinics. The study was approved by the institutional ethical committee and all participants were provided with written informed consent. Details on the characteristics of the participants and study protocol were previously described [21]. Briefly, eligible subjects were men and women, aged from 18 to 75 years, with a body mass index (BMI) ranging from 25 to 40 kg/m^2 ^and pre-diabetes (impaired glucose tolerance or impaired fasting glucose). Entry criteria required triglyceride levels ≤ 350 mg/dL and LDL-cholesterol ≤ 200 mg/dL, stable blood pressure and no evidence of cardiovascular, hepatic or renal diseases. Subjects were not taking anti-inflammatory agents or others interfering with lipid or glucose metabolism. Eligible participants were recruited from June 2005 to May 2006.

In this open-label uncontrolled clinical trial, 290 subjects with weight excess, with or without family history of diabetes, were screened for the interventional protocol and 50 with impaired glucose tolerance (IGT) or impaired fasting glucose (IFG) were randomly assigned to 2 groups that would receive ezetimibe 10 mg/day (n = 25) or simvastatin 20 mg/day (n = 25), preceded by a 2-week run-in period. Simple randomization was applied throwing a dice for each participant to define the initial therapy. Monotherapies were maintained for 12 weeks; thereafter the drugs were combined in each group for an additional 12-week period. All participants received individual counseling for a healthy lifestyle and had monthly visits. Treatment was to be discontinued prematurely if transaminases exceeded 3 times the upper limit of reference or creatine phosphokinase (CPK) 10 times, but no subject met these criteria. One of the ezetimibe group dropped out due to non-compliance. Sample size and power to detect differences with the lipid-lowering agents were calculated based on the reduction in LDL-cholesterol concentration. The power to detect differences with the interventions was 0.75. In addition to expected reductions in lipid concentrations, inflammatory markers (CRP, IL-6 and TNF-α) changes were also considered outcomes of the interventions.

Baseline, 12-week and 24-week blood samples were drawn in the morning, after a 12-hour fast, for glucose, lipid profile, including apolipoprotein A-I and B, leukocyte count and inflammatory markers were made. A LDL-cholesterol goal of 100 mg/dL was used in the present study [8,22].

### Laboratory analysis

Plasma glucose, transaminases, CPK and creatinine were determined by routine methods. Serum lipid levels (total cholesterol, HDL-cholesterol, and triglycerides) were analyzed by commercially available tests (Roche Diagnostics GmbH, Mannheim, Germany). Blood samples were stored at -20°C until determinations of apolipoproteins and inflammatory markers. Apolipoprotein A-I and B were measured by immunoturbidimetry (Olympus Life and Material Science Europa GmbH, Lismeeham, Ireland), with an intra-assay coefficient of variability (CV) of 1.26-1.30% and 0.93-1.17% respectively, and an inter-assay CV of 1.43-1.55% and 1.10-1.46%, respectively. High-sensitivity CRP (Immulite - DPC, Los Angeles, CA, USA), TNF-α and IL-6 (Immulite - Euro/DPC, Llanberis, Gwynedd, UK) were determined by chemiluminescent immunometric assay. The sensitivity of CRP assay was 0.01 mg/dL (intra-assay CV 4.2-6.4% and inter-assay CV 4.8-10%), of TNF-α assay 1.7 pg/mL (intra-assay CV 2.6-3.6%, inter-assay CV 4.0-6.5%) and of IL-6 assay was 2.0 pg/mL (intra-assay CV 3.5-6.2%, inter-assay CV 5.1-7.5%).

### Statistical analysis

Data were expressed as mean values and standard errors or deviations. Unpaired Student's *t *test was used to compare groups at baseline and chi-square to assess differences between qualitative data. One-way ANOVA for repeated measures was used to evaluate the effect of drugs over time and to compare data between groups of subjects according to the type of therapy. In such analysis, pairwise contrasts were made by comparing least-square mean estimates; p-values were adjusted for multiple comparisons using the Bonferroni Holm method. A significant p-value (p < 0.05) signalizes the existence of a statistical difference, considering that the alpha value of 5% was divided by the number of comparisons for each variable. If a difference in a given variable was detected along the time, 3 comparisons (baseline *versus *monotherapy, baseline *versus *combination and monotherapy *versus *combination) were performed, considering that the effect was significant when p values was < 0.05 divided by 3 (significant p value < 0.017). Correlation between variables was tested by the Pearson coefficient. This coefficient was also employed to assess whether changes in variables over time were correlated. Data analysis was performed using Statistical Analysis System software, version 8.2 (SAS Institute, Cary, NC).

## Results

Main baseline characteristics of the subjects are described in Table 1. Except for higher mean fasting plasma glucose for the group which started monotherapy with simvastatin, groups were similar regarding sociodemographic data, frequency of hypertension, mean values of blood pressure and biochemical variables.

**Table 1:** Baseline characteristics of the participants.

	Ezetimibe n = 24	Simvastatin n = 25	p-value
Women (%)	79	76	
Caucasians (%)	41	47	
Smoking (%)	4	12	
Hypertension (%)	92	80	
Age (years)	53.4 ± 9.3	53.1 ± 8.1	0.893
Body mass index (kg/m^2^)	33.1 ± 4.5	31.9 ± 3.4	0.333
Systolic blood pressure (mmHg)	129.3 ± 16.2	124.0 ± 20.4	0.325
Diastolic blood pressure (mmHg)	83.3 ± 8.7	81.6 ± 11.5	0.575
Total cholesterol (mg/dL)	237.4 ± 43.2	214.4 ± 39.7	0.058
LDL-cholesterol (mg/dL)	145.9 ± 39.9	129.4 ± 36.8	0.139
HDL-cholesterol (mg/dL)	56.1 ± 13.7	53.1 ± 11.5	0.408
Triglycerides (mg/dL)	176.8 ± 85.4	160.0 ± 65.5	0.443
Fasting plasma glucose (mg/dL)	104.3 ± 6.7	110.0 ± 11.7	0.041

Data are expressed in number of subjects or mean ± standard deviation.

Only for those subjects who started the protocol with ezetimibe (ezetimibe group), did the mean values of BMI and abdominal circumference decrease significantly after monotherapy, but not following the combination therapy (Table 2). No variation in these parameters was observed for the simvastatin group.

**Table 2:** Anthropometry, biochemical and inflammatory variables in different moments of the study protocol.

	Baseline	Monotherapy	Combination	p value
**Ezetimibe group**				
Body mass index (kg/m^2^)	33.0 ± 0.9	32.4 ± 0.9*	32.4 ± 1.0*	0.002
Abdominal circumference (cm)	102.9 ± 2.1	100.6 ± 2.2*	101.4 ± 2.2	0.010
Total cholesterol (mg/dL)	237.4 ± 8.8	197.0 ± 7.2*	147.8 ± 6.4*#	<0.001
LDL-cholesterol (mg/dL)	145.9 ± 8.2	112.5 ± 6.4*	66.6 ± 4.8*#	<0.001
HDL-cholesterol (mg/dL)	56.1 ± 2.8	55.4 ± 2.1	56.9 ± 2.5	0.855
Triglycerides (mg/dL)	176.8 ± 17.4	145.4 ± 15.9*	121.4 ± 11.3*	<0.001
Apolipoprotein B (mg/dL)	115.6 ± 5.0	93.3 ± 4.6*	66.9 ± 3.7*#	<0.001
Apolipoprotein A-I (mg/dL)	157.7 ± 5.3	153.2 ± 5.0	151.1 ± 6.3	0.149
Leucocytes (×10^3 ^cel/mm^3^)	5.83 ± 0.26	6.07 ± 0.25	6.00 ± 0.31	0.394
C reactive protein (mg/dL)	0.57 ± 0.12	0.46 ± 0.09	0.44 ± 0.14	0.452
Interleukin-6 (pg/mL)	3.1 ± 0.5	2.5 ± 0.3	2.5 ± 0.2	0.286
TNF-α (pg/mL)	8.8 ± 3.2	5.9 ± 0.3	6.1 ± 0.2	0.229
**Simvastatin group**				
Body mass index (kg/m^2^)	31.9 ± 0.7	31.8 ± 0.7	31.6 ± 0.8	0.660
Abdominal circumference (cm)	101.8 ± 1.6	101.8 ± 1.6	101.3 ± 1.9	0.999
Total cholesterol (mg/dL)	214.4 ± 7.9	168.1 ± 8.3*	139.5 ± 7.0*#	<0.001
LDL-cholesterol (mg/dL)	129.4 ± 7.4	84.8 ± 7.0*	62.4 ± 5.5*#	<0.001
HDL-cholesterol (mg/dL)	53.1 ± 2.3	53.2 ± 2.4	54.5 ± 2.5	0.885
Triglycerides (mg/dL)	160.0 ± 13.1	150.5 ± 17.3	112.6 ± 9.7*#	<0.001
Apolipoprotein B (mg/dL)	103.8 ± 4.8	77.9 ± 4.5*	63.3 ± 4.0*#	<0.001
Apolipoprotein A-I (mg/dL)	154.9 ± 3.8	152.1 ± 4.2	152.5 ± 4.7	0.375
Leucocytes (×10^3 ^cel/mm^3^)	5.91 ± 0.36	5.87 ± 0.40	5.91 ± 0.44	0.924
C reactive protein (mg/dL)	0.59 ± 0.14	0.48 ± 0.12	0.35 ± 0.12*#	0.023
Interleukin-6 (pg/mL)	2.8 ± 0.3	2.3 ± 0.2	2.5 ± 0.2	0.146
TNF-α (pg/mL)	6.1 ± 0.2	6.2 ± 0.2	6.6 ± 0.4	0.261

Data are expressed in means ± standard error.

* p < 0.017 *versus *baseline

^# ^p < 0.017 *versus *monotherapy

**Figure 1:**
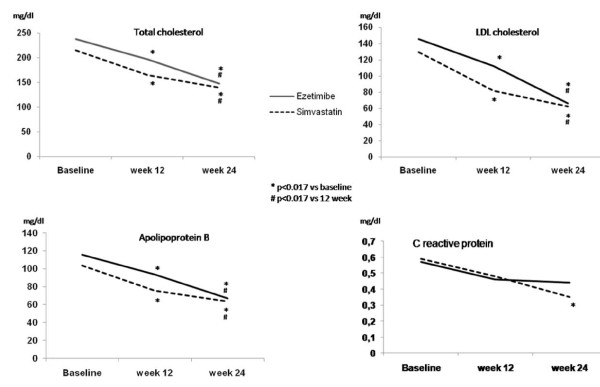
**Lipid profile and C reactive protein concentration of subjects initially receiving ezetimibe and simvastatin at baseline, on monotherapy and with combined therapy**.

Total cholesterol, LDL-cholesterol and apolipoprotein B had similar profiles during follow-up for both groups (Figure 1). Significant declines were observed in all the periods analyzed, as shown in Table 2. A combination of simvastatin and ezetimibe was more effective than isolated drugs in reducing lipid levels. However, simvastatin in isolation showed a greater improvement in total cholesterol, LDL-cholesterol and apolipoprotein B than did ezetimibe monotherapy. Thirty-five percent of the subjects treated with ezetimibe monotherapy reached the goal of 100 mg/dl, as did 72% with simvastatin monotherapy, and 91% with a combination of drugs. Similar percent increments were verified when apolipoprotein B levels were considered. Significant decreases in triglyceride levels were found with the combination therapy, but no significant changes were observed with monotherapies. HDL-cholesterol or apolipoprotein A-I concentrations did not change throughout the whole protocol.

The effects of ezetimibe and simvastatin monotherapies and of the combination of drugs on inflammatory parameters are shown in Table 2. Decreases in CRP and IL-6 induced by ezetimibe therapy were not significant. In the simvastatin group, CRP reduces significantly and the decline was accentuated following the combined therapy with ezetimibe. The changes in CRP showed no correlation with changes in LDL-cholesterol levels.

As far as inflammatory markers are concerned, baseline values of CRP levels were positively correlated to leukocyte count (r = 0.55, p < 0.001), IL-6 (r = 0.25, p < 0.05), TNF-α (r = 0.24, p = 0.05), and abdominal circumference (r = 0.24, p = 0.05). Leukocyte count was also correlated to abdominal circumference (r = 0.27, p < 0.05). Table 2 shows that both drugs induced reductions in CRP, reaching statistical significance only after the addition of ezetimibe to the simvastatin therapy (Figure 1). However, no significant change was observed in mean levels of TNF-α, IL-6 and leukocyte count throughout the whole study. A significant higher number of subjects treated with the combination of ezetimibe and simvastatin achieved a CRP concentration less than 0.2 mg/dL when compared with those undergoing simvastatin monotherapy (64% vs. 48%, respectively, p < 0.05).

## Discussion

This study provides further proof of the role of the combination of 2 different classes of lipid-lowering agents in improving the atherogenic lipid profile of pre-diabetic subjects. The combination of simvastatin and ezetimibe resulted in greater reductions in LDL-cholesterol, apolipoprotein B and triglyceride levels when compared to both monotherapies. The long-term control of lipid profile has been consistently associated with a decrease in cardiovascular morbidity and mortality of subjects with or without glucose metabolism disturbances [1-3,10,11], but more recent evidence suggests that cardiovascular protection may be also attributed to the attenuation of low grade inflammation [5-7]. Considering that glucose intolerance is a major cardiovascular risk factor and a pro-inflammatory condition, a therapeutic approach able to reduce lipids and inflammatory markers should be of particular interest [23].

Statins are the most important lipid-lowering agents used in clinical practice for patients at different levels of cardiovascular risk. Despite their efficacy, almost fifty percent of at-risk patients do not achieve LDL-cholesterol goals according to the European Second Joint Task Force and the US National Cholesterol Education Program Adult Treatment Panel III guidelines [8,22,24]. Since monotherapy may be ineffective in reaching the target, more options are desirable to optimize the management of hypercholesterolemic subjects. In the present study, the percentage of those reaching a LDL-cholesterol goal of 100 mg/dl increased from 72% induced by statin monotherapy to 91% following the simvastatin-ezetimibe combination.

In addition to the improvement in lipid profile, synergistic anti-inflammatory effects of the 2 agents used in the present study are suggested by our findings on serum concentrations of CRP, an established inflammatory marker associated with cardiovascular risk [25,26]. It is known that the beneficial effects of statins on cardiovascular system go beyond decreasing cholesterol levels. Atherosclerotic process should also be delayed by diminishing inflammation, which is translated into lower inflammatory markers, better anti-thrombotic activity and endothelial function. However, it was not demonstrated whether anti-inflammatory effects of statins *per se *would help to postpone diabetes mellitus development.

Some investigators have proposed that CRP concentration may be the strongest predictor of cardiovascular events when compared to other risk factors, but this is still a matter of controversy [27,28]. Achieving lower levels of CRP with lipid-lowering agents may have an additional benefit on the cardiovascular system of subjects at elevated risk such as pre-diabetic ones. Subjects included in the present study had normal or slight elevation of cholesterol levels. It was not known if the use of statin to reduce CRP in normocholesterolemic subjects was able to improve cardiovascular outcomes. Recently, an analysis of 15,548 initially healthy men and women participating in the JUPITER trial corroborated this statement [29]. CRP concentration was predictive of cardiovascular event rates irrespective of the lipid endpoint use. The most benefits were found in those participants who achieved normal LDL-cholesterol and CRP < 1 mg/L.

Ezetimibe added to simvastatin therapy provoked a significant improvement in CRP levels and such an effect was independent of LDL-cholesterol decrease. The ability of statin therapy to reduce CRP has been reported, but a synergistic effect of the combination with ezetimibe has been less frequently described [17-20]. Sager *et al *have demonstrated that ezetimibe 10 mg plus simvastatin is more efficient in lowering the serum concentration of CRP than simvastatin in isolation, in all doses analyzed (10 till 80 mg) independently of the LDL-cholesterol fall [17,18]. In the VYTAL study [19], the fixed combination ezetimibe/simvastatin reduced CRP more than atorvastatin monotherapy. Taking into consideration all the doses used, the combination achieved both LDL and CRP goals in a markedly higher number of patients than did atorvastatin monotherapy. In our study, more subjects treated with the combination of ezetimibe and simvastatin achieved a CRP value less than 0.2 mg/dl when compared with those undergoing simvastatin monotherapy (64% vs. 48%, respectively). In agreement, Pearson *et al *[20], using the same fixed combination of ezetimibe/simvastatin 10/20 mg, found a similar decrease of 30% in CRP levels.

Few studies are available to investigate the attenuation of pro-inflammatory status based on leukocytes count and IL-6 and TNF-α levels. Some studies suggest that statins are able to reduce IL-6 and TNF-α levels [23,30]. In a randomized, double-blind, placebo-controlled clinical trial, conducted on 50 subjects, daily administration of 40 mg of simvastatin provoked significant decreases in IL-6 and TNF-α concentrations [23]. In another study, our group found that a lower dose of simvastatin (20 mg/day), but over a longer period (16 weeks), was able to decrease IL-6 levels significantly [30]. As far as we know, no study focusing on the responses of these inflammatory markers to the combination of statin and ezetimibe is available. In the present study, no change was detected regarding IL-6 or TNF-α levels and leukocytes count.

A weakness of our study is the sample size. The relative small sample size associated with the large range of variation observed in these parameters may have limited to detect statistical differences. In fact, our sample size was not calculated for comparisons between the inflammatory markers. In addition, a lower statin dose and shorter follow-up period could be contributing to the lack of IL-6 and TNF-α responses in our study. Another explanation could be based on the fact that IL-6 and TNF-α actions are mainly at autocrine and paracrine levels [31] and circulating measurements of cytokines may be inappropriate to detect variations at those sites.

In summary, we have confirmed the synergistic lowering effect of low-to-moderate simvastatin and ezetimibe combination on LDL-cholesterol, apolipoprotein B and triglycerides levels in pre-diabetic subjects. As far as inflammatory markers are concerned, a favorable effect, independently of LDL-cholesterol change, is suggested by the CRP reduction, but not by other parameters. Other prospective studies, including larger samples and higher medication doses, may be necessary to draw conclusions about the role of statins and ezetimibe combination on pro-inflammatory profile and long-term benefits for cardiometabolic risk.

## Competing interests

The authors declare that they have no competing interests.

## Authors' contributions

ALAK raised the literature, selected the patients, conducted the study protocol, performed the statistical analysis, and participated in writing the manuscript.

MCB made improvements in the study design, participated in the analysis and discussion of the results.

SRGF raised the hypothesis, conceived of the study, developed the study protocol, helped with the selection of patients, participated in data analysis and writing the manuscript.

All authors read and approved the final manuscript.
